# Malnutrition, a new inducer for arterial calcification in hemodialysis patients?

**DOI:** 10.1186/1479-5876-11-66

**Published:** 2013-03-18

**Authors:** Kun Zhang, Gang Cheng, Xue Cai, Jie Chen, Ying Jiang, Tong Wang, Jingfeng Wang, Hui Huang

**Affiliations:** 1 Department of Cardiology, Sun Yat-sen Memorial Hospital of Sun Yat-sen University, 107 West Yanjiang Road, Guangzhou, 510120, China; 2 Guangdong Province Key Laboratory of Arrhythmia and Electrophysiology, Guangzhou, 510120, China; 3 Plastic and reconstructive surgery department, the First Affiliated Hospital of Sun Yat-sen University, Guangzhou, 510080, China; 4 Department of nephrology, the second hospital of Nanchang, Nanchang, 330003, China; 5 Department of Radiation Oncology, Sun Yat-sen Memorial Hospital of Sun Yat-sen University, Guangzhou, 510120, China; 6 School of Mathematics and Computational Science, Sun Yat-sen University, Guangzhou, 510275, China

**Keywords:** Arterial calcification, Hemodialysis, Malnutrition, Bone morphogenetic protein 2, Matrix Gla protein

## Abstract

**Background:**

Arterial calcification is a significant cardiovascular risk factor in hemodialysis patients. A series of factors are involved in the process of arterial calcification; however, the relationship between malnutrition and arterial calcification is still unclear.

**Methods:**

68 hemodialysis patients were enrolled in this study. Nutrition status was evaluated using modified quantitative subjective global assessment (MQSGA). Related serum biochemical parameters were measured. And the radial artery samples were collected during the arteriovenous fistula surgeries. Hematoxylin/eosin stain was used to observe the arterial structures while Alizarin red stain to observe calcified depositions and classify calcified degree. The expressions of bone morphogenetic protein 2 (BMP2) and matrix Gla protein (MGP) were detected by immunohistochemistry and western blot methods.

**Results:**

66.18% hemodialysis patients were malnutrition. In hemodialysis patients, the calcified depositions were mainly located in the medial layer of the radial arteries and the expressions of BMP2 and MGP were both increased in the calcified areas. The levels of serum albumin were negatively associated with calcification score and the expressions of BMP2 and MGP. While MQSGA score, serum phosphorus and calcium × phosphorus product showed positive relationships with calcification score and the expressions of BMP2 and MGP.

**Conclusions:**

Malnutrition is prevalent in hemodialysis patients and is associated with arterial calcification and the expressions of BMP2 and MGP in calcified radial arteries. Malnutrition may be a new inducer candidate for arterial calcification in hemodialysis patients.

## Background

Arterial calcification is a major risk factor of cardiovascular mortality, particularly for hemodialysis patients [1]. It increases arterial stiffness, pulse wave velocity, decreases arterial compliance and ultimately leads to severe cardiovascular events [2,3].

Arterial calcification is traditionally considered to be a passive process; however, recent studies have shown that arterial calcification is an actively regulated process and a series of factors are involved in this process [4]. Bone morphogenetic protein 2 (BMP2) and matrix Gla protein (MGP) are two closely linked critical proteins that regulate arterial calcification [5]. And BMP2 has been found to be a promoter for arterial calcification while MGP is considered to be an inhibitor of arterial calcification [6,7]. However, it is still not clear, which key factor associated with the prevalence of arterial calcification and expressions of BMP2 and MGP in end-stage renal disease (ESRD).

Malnutrition is also a pivotal factor associated with the increased morbidity and mortality in hemodialysis patients [8]. However, whether malnutrition participates in the process of arterial calcification and associates with the expressions of BMP2 and MGP are not well understood. In the present study, we estimated the nutrition status of hemodialysis patients and investigated the role of malnutrition in the process of arterial calcification.

## Materials and methods

### Patients

All 68 hemodialysis patients from March 2010 to January 2012 were eligible for the study. The inclusion criteria were as follows: all patients had begun to maintaining hemodialysis. In this study most patients had been dialyzed for at least 3 months, except two subjects dialyzed for 1 month. Hemodialysis criteria: sustained creatinine clearance rate (Ccr) <10 ml/min, serum creatinine (Scr) > or =707 μmol/L and manifestations of the clinical syndromes such as fatigue, uneasy, gastrointestinal syndrome, anemia, and acidosis. The patients with acute renal failure, gastrointestinal cancers, hematological system diseases, primary parathyroid diseases and severe infections were excluded. We also excluded the patients with primary bone diseases, but not the typical bone disease in patients with chronic renal failure, “renal osteodystrophy”. And the patients that had been receiving a blood transfusion or intravenous albumin within one month before nutrition assessment were also excluded. The radial artery samples from normal nutrition and malnutrition hemodialysis patients were collected during the arteriovenous fistula surgeries in Sun Yat-sen University. The study protocol conformed to the ethical guidelines of the 1975 Declaration of Helsinki as reflected in a priori approval by the Ethics Committee of Sun Yat-sen University. Informed consent was obtained from each patient.

### Demographic characteristic data collection and nutritional status evaluation

Demographic characteristic data were collected as follows: gender, age, height, weight, primary diseases, complications and hemodialysis duration.

Modified quantitative subjective global assessment (MQSGA), a good method for assessing the nutritional status, was specially used for ESRD patients. In this study, we used MQSGA for assessing the nutritional status of the enrolled hemodialysis patients. MQSGA consists of seven components: weight change, gastrointestinal symptoms, dietary intake, functional capacity, co-morbidity, subcutaneous fat and signs of muscle wasting. Each component has a score from 1 (normal) to 5 (very severe). The ‘malnutrition score’ is a number between 7 (normal) and 35 (severe malnourished) [9]. Based on the MQSGA score, two groups were classified: normal nutrition group (score of 7–10) and malnutrition group (score of 11–35).

### Biochemical data collection

Before operation, blood samples were collected into tubes containing ethylenediaminetetraacetic acid (EDTA) and then stored at −20°C before being analyzed. Serum calcium, serum phosphorus, albumin, triglyceride, total cholesterol (TG), low density lipoprotein cholesterol (LDL-c), and high density lipoprotein cholesterol (HDL-c) were measured using TBA-120 auto-analyzer (Toshiba Medical Systems, Japan).

Serum levels of intact parathyroid hormone (PTH) were assessed by the chemiluminescence method (Immulyte 2000; DPC, Los Angeles, CA). The calcium × phosphorus (Ca × P) was calculated based on the serum calcium and serum phosphorus. Serum calcium 1 mmol/L =4 mg/dl, serum phosphorus 1 mmol/L =3.1 mg/dl, adjusted serum calcium (mg/dl): serum calciculm +0.8× [4-sAlb (serum albumin) (g/dl)].

### Histological analysis

A 2–3 mm circumferential segment of radial artery was excised from the patients during arteriovenous fistula surgeries and immediately placed in saline. The samples were dissected free of fat and subcutaneous, fixed overnight in 4% paraformaldehyde and then embedded in paraffin. Tissue sections of 4 μm thickness were prepared and stained with hematoxylin/eosin (HE, Beyotime institute of Biology, Suzhou, China). And Alizarin red (Genmed Scientifics Inc. USA).

### HE stain

3 to 5 sections per specimen were deparaffinized and hydrated to water. Stain nuclei with haematoxylin for 10 minutes. Then rinse in running tap water for 10 minutes. After dehydrate 5 seconds in 95% alcohol, the sections were counterstained with eosin from 30 seconds to 2 minutes depending on the age of the eosin, and the depth of the counterstain desired. Dehydrate in 95% alcohols 2 minutes and repeat one time to remove the excess eosin. Under the microscope, we could see the nuclei were stained in blue while cytoplasm was stained in pink.

### Alizarin red stain

Alizarin red stain is a PH dependent method for differentiating calcium oxalate from calcium carbonate and phosphate. 3 to 5 sections per specimen were deparaffinized and hydrated to distilled water. Stain slides with the Alizarin Red Solution for about 2 minutes. Shake off excess dye and blot sections. Dehydrate in acetone and then in Acetone-Xylene (1:1) solution. Clear in xylene and mount in a synthetic mounting medium. Under the microscope, we could see the calcium deposits (except oxalate) were stained in orange-red.

### Histological calcification score

The histological calcification score was obtained by averaging all the scores from all sections of Alizarin red stain and graded from 0 to 4 [10]. A score of <1 was labeled “no calcification”, score of 1 to 2.5 = mild/moderate calcification and >2.5 to 4 = severe calcification. According to the Alizarin red stain score, we classified the patients into three groups: non-calcified (NC) group, slight calcified (SLC) group and severe calcified (SEC) group.

### Immunohistochemical stain

3 to 5 unstained slides of each tissue section were deparaffinized in xylene and rehydrated in descending achohol. Slides were then placed in 3% hydrogen peroxide to inhibit endogenous peroxidase. After washing in Tris saline, the sections were blocked by 3% bovine serum albumin (Sigma-Aldrich Tradign Co, Ltd) for 15 minutes, and then incubated with primary antibody at appropriate dilutions overnight. The antibodies utilized were: BMP2 (1:100, Abcam) and MGP (1:100, Santa Cruz Biotechnology). Staining without the primary antibody severed as a negative control. The second antibody peroxide conjugated goat-anti-mouse (Weijia, Guangzhou, China) at 1:400 dilution was applied for 45 minutes, developed with DAB (Weijia, Guangzhou, China), and the sections counterstained with Harris Hematoxylin (Weijia, Guangzhou, China).

### Immunohistochemistry analysis

The expressions of BMP2 and MGP were semiquantitative analyzed with Image Pro plus 6.0 digital analysis systems (Media Cybernetics, USA). Under light microscope, the mean optical density value (MODV) of the cells was calculated at 5 visual fields randomly. MODV represented the density of positive proteins. The mean value of the measured value was used as the final value for calculation.

### Western blot analysis

Radial artery tissues were homogenized in 250 μ of homogenization buffer using an electronic stirrer. Protein concentration was determined with BCA kit (Biocolors, Shanghai, China).

Equal amounts (30 μg) of total protein were then separated by electrophoresis on polyacrylamide-SDS gels and transferred onto a polyvinylidene difluoride membrance (immobilon-P membranes; Millipore, Bedford, MA) using the wet method. The membranes were washed and blocked with 5% nonfat dry milk in TBS/T buffer for 1 hour at room temperature. Then, the membranes were incubated overnight at 4°C with primary antibodies: mouse anti-BMP2 (1:1000) and mouse anti-MGP (1:1000), and mouse anti-β-actin (1:1000, Weijia, Guangzhou, China). After washing, membranes were incubated with goat peroxidase-conjugated secondary antibody (1:5000, Weijia, Guangzhou, China) and then visualized with an ECL kit (Thermo Scientific, USA) according to the instructions. Specific bands were quantified by Quantity One software (Bio-Rad, USA).

### Statistical analysis

Data are expressed as mean ± standard error (SEM). Since not each variable was normally distributed, Pearson coefficient correlation and Spearman Rank Correlation were used to assess the relationship among calcification score, expressions of BMP2 and MGP, MQSGA score and several calcification-related risk factors. Multiple linear regression analyses were utilized to estimate the factors that affected arterial calcification. The ANOVA test and Kruskal-Wallis test were used for comparing biochemical characteristics and MQSGA score among different calcified groups. P value <0.05 was considered statistically significant. All analyses were performed using SPSS version 17 (SPSS, Inc, Chicago IL).

## Results

### Demographic and biochemical characteristics in hemodialysis patients

A total of 68 patients (35 male and 33 female) undergoing hemodialysis were included in this study. The mean age of the patients was 62 ± 2.15 years old. Nearly each patient (92.31%) had a history of hypertension while about one third (30.77%) patients suffered diabetes. The mean hemodialysis duration was 5.6 months. We grouped the patients depending on the MQSGA score. 45 patients (66.18%) were malnutrition. Demographic and biochemical characteristics of the two groups were presented in Table 1. Comparing with the normal nutrition group, the malnutrition group showed significantly lower body weight (56.79 ± 1.56 kg *vs.* 61.93 ± 1.76 kg, p = 0.046) and body weight index (BMI) (21.18 ± 0.46 g/m^2^*vs.* 23.00 ± 0.54 g/m^2^, p = 0.018) and a significantly higher MQSGA score (25.28 ± 1.13 *vs.* 8.26 ± 1.73, p < 0.001). As for the main biochemical parameters, the malnutritional patients had high levels of adjusted serum calcium, serum phosphorus, Ca × P, PTH, HLD-c, and low levels of serum albumin (Table 1).

**Table 1:** Demographic and biochemical characteristics in hemodialysis patients

	**Normal nutrition (n = 23)**	**Malnutrition (n = 45)**	**P**
Age (year)	63.43 ± 2.68	62.22 ± 2.26	0.74
Sex ratio (male/female)	13/10	22/23	0.83
Height (m)	1.64 ± 0.06	1.63 ± 0.67	0.99
Weight (kg)	61.93 ± 1.76	56.79 ± 1.56	0.046
BMI (g/m^2^)	23.00 ± 0.54	21.18 ±0.46	0.018
MQSGA	8.26 ± 1.73	25.28 ± 1.13	<0.001
Ca (mmol/L)	1.99 ± 0.041	2.10 ± 0.023	0.055
Ad-Ca (mg/dL)	8.13 ± 0.21	8.93 ± 0.15	<0.001
P (mmol/L)	1.21 ± 0.13	1.80 ± 0.11	0.0070
Ca × P (mg²/dL²)	44.67 ± 2.53	49.00 ± 2.82	0.035
PTH (pg/mL)	158.50 ± 20.44	535.10 ± 95.19	0.026
Alb (g/L)	37.63 ± 1.02	33.23 ± 1.45	<0.001
TG (mmol/L)	2.88 ± 0.75	1.85 ± 0.16	0.51
Chol (mmol/L)	3.40 ± 0.25	4.05 ± 0.15	0.78
LDL-c (mmol/L)	2.31 ± 0.21	2.34 ± 0.19	0.093
HDL-c (mmol/L)	1.28 ± 0.25	1.83 ± 0.15	0.013

Values are given as mean ± SEM. Nutritional status was classified into two groups according to MQSGA score: normal nutrition group and malnutrition group.

Ad-Ca, albumin adjusted serum calcium; Alb, albumin; BMI, body mass index; Ca × P, calcium × phosphorus product; Chol, cholesterone; HDL-c, high-density lipoprotein cholesterol; LDL-c, low-density lipoprotein-cholesterol; MQSGA, modified quantitative subjective global assessment; P, phosphorus; PTH, parathyroid hormone; TG, triglyceride.

### Medial calcification located in the radial arteries of hemodialysis patients

**Figure 1:**
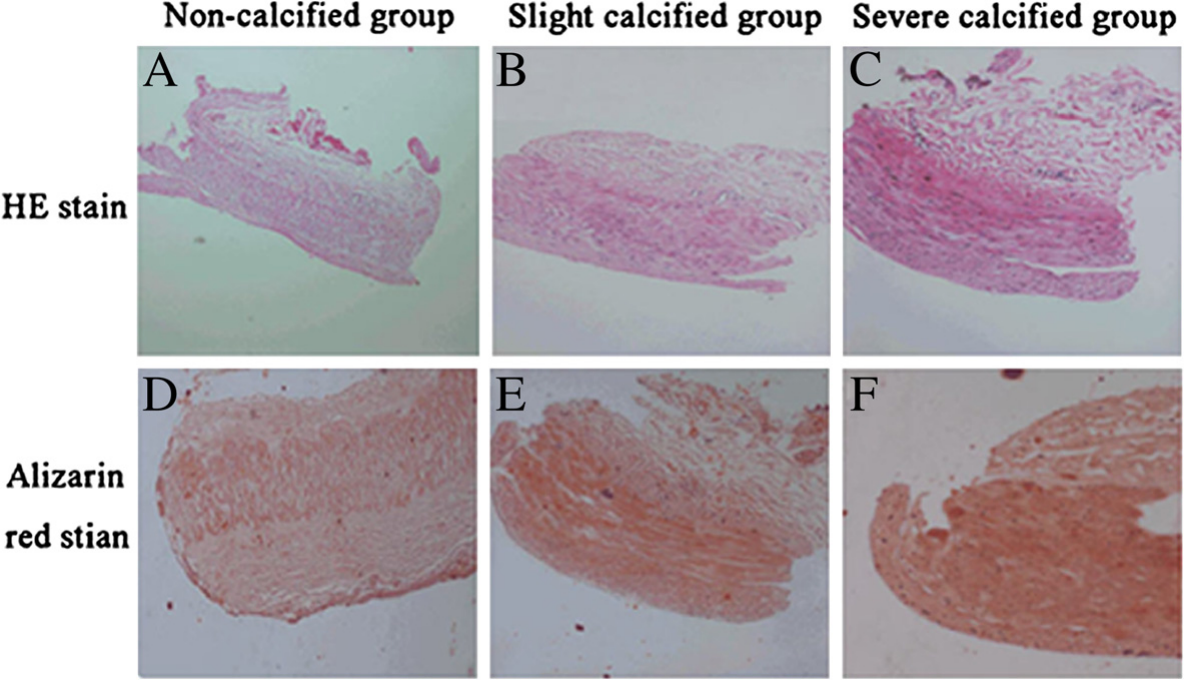
**Hematoxylin/eosin (HE, A-C) and Alizarin red (D-F) stains of radial arteries of malnutritional hemodialysis patients.** HE stain (**A**-**C**) showed that the thickness of medial layer was increased along with the calcified degree. And the Alizarin red stain (**D**-**F**) demonstrated that the calcified depositions were mainly located in the medial layer of the radial arteries. Abbreviate: NC, non-calcified; SLC, slight calcified; SEC, severe calcified.

To observe the structures and calcified depositions in the radial arteries of hemodialysis patients, HE and Alizarin red stains were performed, and the representative data were showed in Figure 1A-F. Alizarin red stain results showed that 46.15% (31/68) of the radial arteries had different degree of calcified depositions and the calcified depositions were mainly located in the medial layer (Figure 1D-F). We grouped the hemodialysis patients based on the Alizarin red stain and calculated the calcification scores depending on the calcified degree of the radial arteries. The mean calcification score was 0.34 for NC group, 1.84 for SLC group and 3.44 for SEC group. In addition, HE stain (Figure 1A-C) also showed that the medial layer of the calcified radial arteries was increased.

### Biochemical characteristics

**Figure 2:**
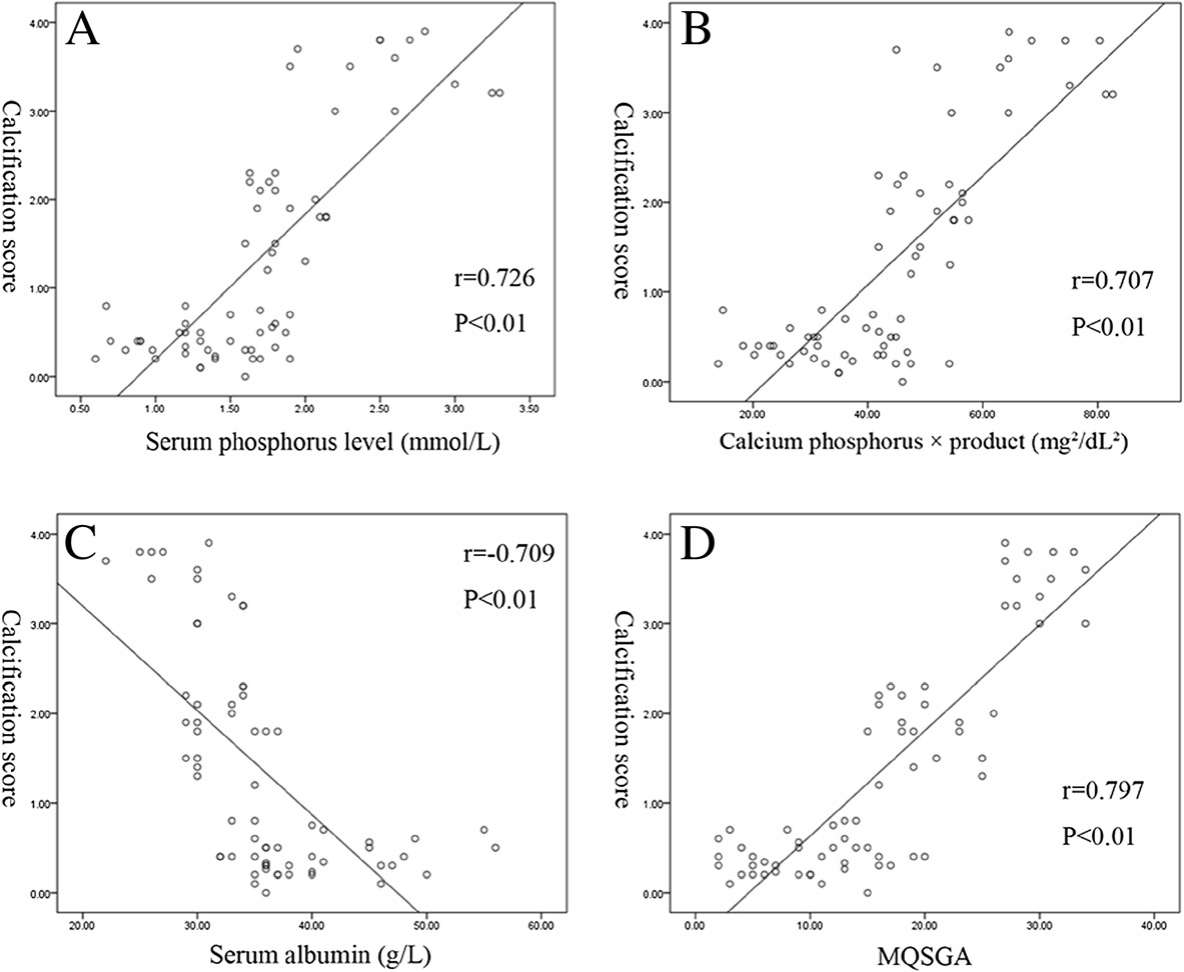
**(A-D) The relationship between calcification score and the levels of serum phosphorus, calcium × phosphorus product, serum albumin and modified quantitative subjective global assessment (MQSGA) score.** The levels of serum phosphorus, calcium × phosphorus product and MQSGA score had a positive relationship with calcification score (Figure 2A, B, D) while the levels of serum albumin showed a negative correlation (Figure 2C).

Table 2 showed the related serum biochemical characteristics and MQSGA scores of the hemodialysis patients. Comparing with NC group, the SLC and SEC groups had significantly elevated levels of serum phosphorus, Ca × P and significantly decreased levels of serum albumin. And accompanied with the calcified degree of radial arteries, the MQSGA score significantly increased. Then we analyzed the relationship between calcified degree and the levels of serum phosphorus, Ca × P and serum albumin. The results indicated that the calcified degree was significantly positively correlated with the levels of serum phosphorus (r = 0.726, p < 0.01) and Ca × P (r = 0.707, p < 0.01), and had a significant negative correlation with the levels of serum albumin (r = −0.709, p < 0.01) (Figure 2A-C). In addition, we also analyzed the relationship between nutritional status and arterial calcification. Figure 2D showed that the MQSGA score had a significantly positive relationship with calcification score (r = 0.797, p < 0.01). And multiple linear regression analyses revealed that MQSGA score was the most important factor affecting calcification score (β = 0.448, p < 0.001) followed by serum phosphorus (β = 0.426, p < 0.001) and serum albumin (β = −0.192, p = 0.008).

**Table 2:** The comparison of biochemical characteristics and MQSGA score among different calcified groups

	**NC (n=37)**	**SLC (n=18)**	**SEC (n=13)**
Ca(mmol/L)	2.03 ± 0.051	2.18 ± 0.023	1.93 ± 0.070
Ad-Ca (mg/dL)	9.20 ± 0.14	9.43 ± 0.19	9.25 ± 0.24
P(mmol/L)	1.34 ± 0.22	1.85 ± 0.15^a^	2.28 ± 0.33^b,c^
Ca × P(mg^2^/dL^2^)	34.12 ± 1.65	50.50 ± 1.23^a^	66.97 ± 3.25^b,c^
PTH (pg/mL)	66.40 ± 2.75	70.93 ± 7.82	77.50 ± 6.00
Alb(g/L)	39.81 ± 1.02	32.11 ± 0.64^a^	29.08 ± 1.02^b^
TG (mmol/L)	2.22 ± 0.82	2.03 ± 0.53	1.79 ± 0.48
Chol (mmol/L)	3.65 ± 0.32	4.10 ± 0.35	4.09 ± 1.01
LDL-c(mmol/L)	2.47 ± 0.27	2.06 ± 0.27	2.23 ± 1.55
HDL-c(mmol/L)	1.47 ± 0.37	2.04 ± 0.39	1.86 ± 0.54
MQSGA	10.79 ± 0.42	20.24 ± 0.85^a^	31.22 ± 1.14^b,c^

Values are given as mean ± SEM. Three groups are classified according to calcification score: non-calcified (NC) group, slight calcified (SLC) group and severe calcified (SEC) group.

Ad-Ca, albumin adjusted serum calcium; Alb, albumin; Ca × P, calcium × phosphorus product; Chol, cholesterone; HDL-c, high-density lipoprotein cholesterol; LDL-c, low-density lipoprotein-cholesterol; MQSGA, modified quantitative subjective global assessment; P, phosphorus; PTH, parathyroid hormone; TG, triglyceride.

^a^ p < 0.05 SLC *vs.* NC; ^b^ p < 0.05 SEC *vs.* NC;^c^ p < 0.05 SEC *vs.* SLC.

### Increased expressions of BMP2 and MGP in calcified radial arteries

**Figure 3:**
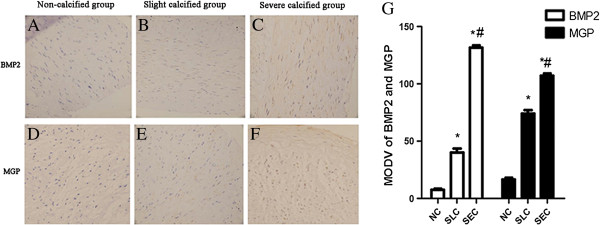
**(A-G) The immunostaining results of bone morphogenetic protein-2 (BMP2) and matrix Gla protein (MGP).** Both slight calcified (SLC) group and severe calcified (SEC) group had significant higher expressions of BMP2 and MGP in radial arteries of hemodialysis patients than non-calcified (NC) group (Figure 3A-G). And comparing with SLC group, the SEC group also showed significantly increased expressions of BMP2 and MGP (Figure 3B-C, E-G).

**Figure 4:**
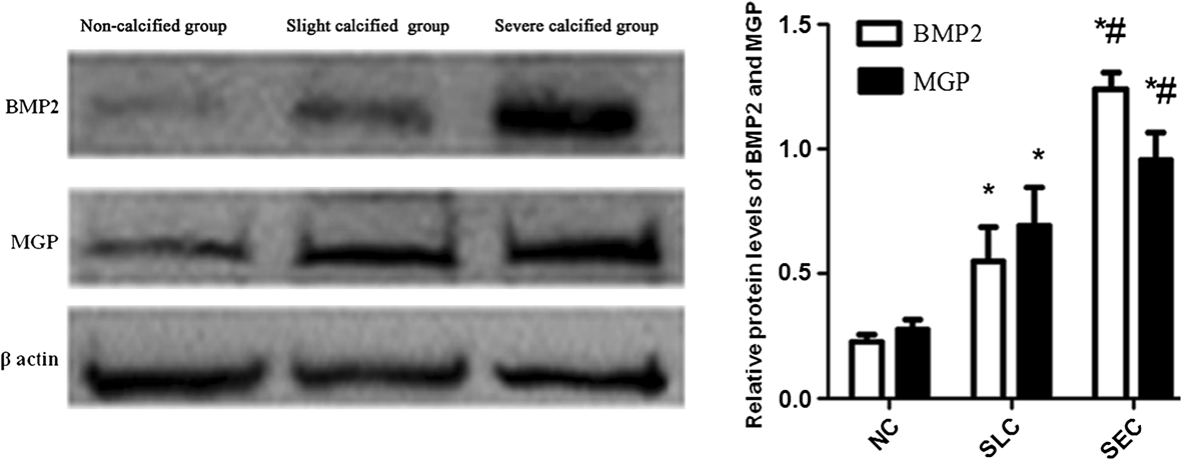
**The western blot results of bone morphogenetic protein-2 (BMP2) and matrix Gla protein (MGP).** Comparing with non-calcified (NC) group, both slight calcified (SLC) group and severe calcified (SEC) group had significantly increased expressions of BMP2 and MGP in radial arteries of hemodialysis patients. And SEC group also had significant higher expressions of BMP2 and MGP than SLC group.

BMP2 and MGP are two critical proteins regulating the process of arterial calcification. The immunostaining of both proteins was almost exclusively in the media layer and prominent in the calcified areas (Figure 3A-F). The MODV which represented the density of positive proteins showed that both the expressions of BMP2 and MGP were gradually significantly increased responding to the calcified degree (Figure 3G). The western blot results also demonstrated that the expressions of BMP2 and MGP significantly increased with the calcified degree (Figure 4). And the ratio of MGP/BMP2 in NC, SLC, and SEC group was 1.27, 1.2, and 0.78 respectively.

### The relationships between BMP2/MGP and serum phosphorus, calcium × phosphorus product, serum albumin and MQSGA score

We explored the relationships between the expressions of BMP2 and MGP and calcification-related factors. Table 3 showed that both the levels of serum phosphorus and calcium × phosphorus product positively correlated with the expressions of BMP2 and MGP in radial arteries of the hemodialysis patients. In contrast, the levels of serum albumin significantly negatively correlated with the expressions of BMP2 and MGP. Moreover, it was showed that the expressions of BMP2 and MGP had positive relationships with MQSGA score (Table 3).

**Table 3:** The relationship between MODV of BMP2/MGP and several calcification-related risk factors

	**MODV of BMP2**		**MODV of MGP**	
	**r**	**P**	**r**	**P**
Phosphorus (mmol/L)	0.586	<0.001	0.659	<0.001
Ca×P (mg²/dL²)	0.498	<0.001	0.541	<0.001
Albumin (g/L)	-0.586	<0.001	-0.577	<0.001
MQSGA	0.707	<0.001	0.648	<0.001

BMP2, bone morphogenetic protein-2; Ca × P, calcium × phosphorus product; MGP, matrix Gla protein; MODV, mean optical density value; MQSGA, modified quantitative subjective global assessment.

## Discussion

This study demonstrated that in hemodialysis patients, malnutrition is prevalent. And the malnourished patients have a high incidence rate of radial arteries calcification. In addition, the expressions of BMP2 and MGP were increased in the calcified radial arteries. For the first time in hemodialysis patients to our knowledge, we demonstrated that malnutrition (a high MQSGA score and a low level of serum albumin) was closely associated with arterial calcification and the expressions of BMP2 and MGP. Furthermore, the definite risk factors such as the serum phosphorus and Ca × P product were both correlated with the calcified degree similarly to the previous studies [11].

Two types of arterial calcification have been observed: intimal calcification of the atherosclerotic plaques and medial calcification of large elastic arteries and small arterioles [12]. In the ESRD, multiple factors were involved in the development of arterial calcification. Besides traditional risk factors, such as hypertension, hyperlipidemia, diabetes, there are also specific risk factors (hyperphosphatemia, uremic toxins, inflammation, etc.) that associated with arterial calcification. However, whether malnutrition, the important risk factor of hemodialysis, is also a specific risk factor for arterial calcification, it is still unclear. In fact, there is a high incidence rate of malnutrition ranging 18% to 75% in maintaining dialysis patients [13]. And malnutrition is an important predictor of mortality in patients with ESRD [14]. Traditional evaluation of nutritional status can be attained by body weight, BMI, anthropometry, biochemical parameters and subjective global assessment. In this study, besides serum albumin, MQSGA was used, and both of which were good ways to evaluate nutrition status of the hemodialysis patients [9,15]. Malnutrition is not only a decline in protein and calorie intake but follows a decrease in the levels of various nutritional makers, for example, vitamin D and vitamin K, both of which were involved in the process of arterial calcification [16]. From the multiple linear regression analyses, we found that malnutrition was the most important factor affecting arterial calcification. And it indicates that malnutrition may be involved in the process of arterial calcification. Hypoalbuminemia is also a common marker of malnutrition and low levels of serum albumin was considered as key diagnostic criteria for malnutrition,especially in hemodialysis patients [17]. And previous epidemiological studies have shown a strong association between serum albumin and cardiovascular mortality in hemodialysis patients [18]. However, there is still lack of a clear explanation for the role of serum albumin in high incidence rate cardiovascular events of hemodialysis patients. In our study, we found that low levels of serum albumin were significantly negatively correlated with arterial calcification. So the low levels of serum albumin may increase the morbidity and mortality of cardiovascular events by promoting the development of arterial calcification in hemodialysis patients. In addition, the low levels of albumin induce inflammation, inadequate transportation of the excessive calcium, phosphorus, cholesterol, triglyceride, etc. and all these factors could promote arterial calcification. However, because of the limited specific measures of malnutrition and sample size, more prospective and randomized studies are needed.

BMP2 and MGP are two closely linked proteins during the process of arterial calcification. BMP2 belongs to the TGF-β (transforming growth factor β) superfamily of growth factors and has been demonstrated playing a key role in the process of arterial calcification [19,20]. MGP, the vitamin K-dependent protein, binds to BMP2 and acts as an endogenous calcification inhibitor [21]. Since both the BMP2 and MGP participate in the process of arterial calcification, we observed the expressions of BMP2 and MGP in calcified radial arteries of hemodialysis patients. We found the expressions of BMP2 and MGP increased simultaneously in the calcified radial arteries. Meanwhile MQSGA score was positively correlated with the expressions of BMP2. And the levels of serum albumin showed a negative correlation with the expressions of BMP2. This provides indirect evidence that malnutrition has a relationship with arterial calcification.

Interestingly, we also found the increased expressions of MGP did not show a BMP2-inhibited effect for arterial calcification. This may attribute to the ratio of MGP/BMP2 or the forms of MGP. Zebboudj et al. demonstrated that MGP dose-dependently regulated the activity of BMP2 and increased calcification [5,22]. In present study, the ratio of MGP/BMP2 decreased with the calcified degree which indicates that the lower ratio of MGP/BMP2, the more severe arterial calcification develops. Moreover, MGP exists carboxylated and uncarboxylated forms, and that might influence the process of calcification differently. In our study, we found that the expressions of MGP were increased in human calcified radial arteries under hemodialysis condition. Two possible explanations are that the increased MGP may be the uncarboxylated form which showed no effect for inhibiting arterial calcification or only a response to the calcified process but not a primary cause of arterial calcification. However, since lacking of assessment of uncarboxylated MGP, more studies in human and animals are needed to explore the role of MGP in arterial calcification.

## Conclusions

Malnutrition is a possible mechanism of arterial calcification in hemodialysis patients. Effective treatment of malnutrition will be a promising strategy for attenuating arterial calcification and decreasing the morbidity and mortality of cardiovascular events in hemodialysis patients.

## Abbreviations

BMP2: Bone morphogenetic protein-2; Ca × P product: Calcium × phosphorus product; ESRD: End-stage renal disease; HDL-c: High density lipoprotein cholesterol; HE: Hematoxylin/eosin; LDL-c: Low density lipoprotein cholesterol; MGP: Matrix Gla protein; MODV: Mean optical density value; MQSGA: Modified quantitative subjective global assessment; NC: Non-calcified; PTH: Parathyroid hormone; SEC: Severe calcified; SLC: Slight calcified; TG: Total cholesterol

## Competing interests

The authors declare that they have no competing interests.

## Authors’ contributions

KZ wrote the manuscript. HH, KZ, and XC participated in conception and design, and the analyses, and wrote the manuscript. GC, YJ and JC participated in data collection and performed the statistical analysis. TW helped to draft the manuscript. JFW reviewed the manuscript and made final changes. All authors have given their final approval of the version to be published.

## References

[B1] Chertow GM Burke SK Raggi P Sevelamer attenuates the progression of coronary and aortic calcification in hemodialysis patients Kidney Int 2002 62 245 252 12081584 10.1046/j.1523-1755.2002.00434.x

[B2] London GM Guerin AP Marchais SJ Metivier F Pannier B Adda H Arterial media calcification in end-stage renal disease: impact on all-cause and cardiovascular mortality Nephrol Dial Transplant 2003 18 1731 1740 12937218 10.1093/ndt/gfg414

[B3] Blacher J Guerin AP Pannier B Marchais SJ London GM Arterial calcifications, arterial stiffness, and cardiovascular risk in end-stage renal disease Hypertension 2001 38 938 942 11641313 10.1161/hy1001.096358

[B4] Cozzolino M Brancaccio D Gallieni M Slatopolsky E Pathogenesis of vascular calcification in chronic kidney disease Kidney Int 2005 68 429 436 16014020 10.1111/j.1523-1755.2005.00421.x

[B5] Zebboudj AF Imura M Bostrom K Matrix GLA protein, a regulatory protein for bone morphogenetic protein-2 J Biol Chem 2002 277 4388 4394 11741887 10.1074/jbc.M109683200

[B6] Li X Yang HY Giachelli CM BMP-2 promotes phosphate uptake, phenotypic modulation, and calcification of human vascular smooth muscle cells Atherosclerosis 2008 199 271 277 18179800 10.1016/j.atherosclerosis.2007.11.031 PMC3249145

[B7] Luo G Ducy P Spontaneous calcification of arteries and cartilage in mice lacking matrix Gla protein Nature 1997 388 78 81 9052783 10.1038/386078a0

[B8] Kopple JD Nutritional status as a predictor of morbidity and mortality in maintenance dialysis patients ASAIO J 1997 43 246 250 9152503

[B9] Kalantar-Zadeh K Kleiner M Dunne E Lee GH Luft FC A modified quantitative subjective global assessment of nutrition for dialysis patients Kidney Int 1999 14 1732 1738 10.1093/ndt/14.7.1732 10435884

[B10] Moe SM O’Neill KD Duan D Ahmed S Chen NX Leapman SB Fineberg N Kopecky K Medial artery calcification in ESRD patients is associated with deposition of bone matrix proteins Kidney Int 2002 61 638 647 11849407 10.1046/j.1523-1755.2002.00170.x

[B11] Giachelli CM Vascular calcification mechanisms J Am Soc Nephrol 2004 15 2959 2964 15579497 10.1097/01.ASN.0000145894.57533.C4

[B12] Goodman WG Vascular calcification in end-stage renal disease J Nephro 2002 15 Suppl 6 S82 S85 12515378

[B13] Mehrotra R Kopple JD Nutritional management of maintenance dialysis patients: why aren’t we doing better? Annu Rev Nutr 2001 21 343 379 11375441 10.1146/annurev.nutr.21.1.343

[B14] Rambod M Bross R Zitterkoph J Benner D Pithia J Colman S Kovesdy CP Kopple JD Kalantar-Zadeh K Association of Malnutrition-Inflammation Score with quality of life and mortality in hemodialysis patients: a 5-year prospective cohort study Am J Kidney Dis 2009 53 298 309 19070949 10.1053/j.ajkd.2008.09.018 PMC5500250

[B15] Honda H Qureshi AR Heimburger O Barany P Wang K Pecoits-Filho R Stenvinkel P Lindholm B Serum albumin, C-reactive protein, interleukin 6, and fetuin a as predictors of malnutrition, cardiovascular disease, and mortality in patients with ESRD Am J Kidney Dis 2006 47 139 148 16377395 10.1053/j.ajkd.2005.09.014

[B16] Ketteler M Rothe H Kruger T Biggar PH Schlieper G Mechanisms and treatment of extraosseous calcification in chronic kidney disease Nat Rev Nephrol 2011 7 509 516 21769106 10.1038/nrneph.2011.91

[B17] Fouque D Kalantar-Zadeh K Kopple J Cano N Chauveau P Cuppari L Franch H Guarnieri G Ikizler TA Kaysen G Lindholm B Massy Z Mitch W Pineda E Stenvinkel P Trevino-Becerra A Wanner C A proposed nomenclature and diagnostic criteria for protein-energy wasting in acute and chronic kidney disease Kidney Int 2008 73 391 398 18094682 10.1038/sj.ki.5002585

[B18] Mehrotra R Duong U Jiwakanon S Kovesdy CP Moran J Kopple JD Kalantar-Zadeh K Serum albumin as a predictor of mortality in peritoneal dialysis: comparisons with hemodialysis Am J Kidney Dis 2011 58 418 428 21601335 10.1053/j.ajkd.2011.03.018 PMC3159826

[B19] Nakagawa Y Ikeda K Akakabe Y Koide M Uraoka M Yutaka KT Kurimoto-Nakano R Takahashi T Matoba S Yamada H Okigaki M Matsubara H Paracrine osteogenic signals via bone morphogenetic protein-2 accelerate the atherosclerotic intimal calcification in vivo Arterioscler Thromb Vasc Biol 2010 30 1908 1915 20651281 10.1161/ATVBAHA.110.206185

[B20] Ducy P Karsenty G The family of bone morphogenetic proteins Kidney Int 2000 57 2207 2214 10844590 10.1046/j.1523-1755.2000.00081.x

[B21] Proudfoot D Shanahan CM Molecular mechanisms mediating vascular calcification: Role of matrix Gla protein Nephrology 2006 11 455 461 17014561 10.1111/j.1440-1797.2006.00660.x

[B22] Zebboudj AF Shin V Bostrom K Matrix GLA protein and BMP-2 regulate osteoinduction in calcifying vascular cells J Cell Biochem 2003 90 756 765 14587031 10.1002/jcb.10669

